# Carbon stocks and dynamics of different land uses on the Cerrado agricultural frontier

**DOI:** 10.1371/journal.pone.0241637

**Published:** 2020-11-06

**Authors:** Emily Ane Dionizio, Fernando Martins Pimenta, Lucas Barbosa Lima, Marcos Heil Costa

**Affiliations:** Department of Agricultural Engineering, Federal University of Viçosa (UFV), Viçosa, Minas Gerais, Brazil; Areospace Information Research Institute Chinese Academy of Sciences, CHINA

## Abstract

The largest and most dynamic agricultural frontier in Brazil is known as MATOPIBA, an area that covers part of the Cerrado biome. Within this region, Western Bahia stands out as a large producer of soy and cotton. There are no studies that quantify carbon stocks for different land uses and land cover types in Western Bahia, which hinders comprehension of the role of agricultural expansion in carbon dynamics and the development of sustainable agriculture policies. Here, we evaluate how the land use changes in this region have affected the carbon balance in the aboveground biomass (AGB), belowground biomass (BGB), and soil reservoirs. We collected soil samples for areas with different land uses and land cover types to estimate soil carbon stocks (SCS) and combined remote sensing results and modeling techniques to develop a historical reconstruction of spatial patterns of SCS, AGB, and BGB during the period 1990–2018. The replacement of areas from the forest formations class with pasture and rainfed agriculture reduced the 100 cm depth SCS (SCS_100_) by 37.3% (*p* = 0.031) and 30.3% *(p* = 0.053), respectively. By contrast, the conversion of pasture and rainfed agriculture to irrigated agriculture increased SCS_100_ by 34% (*p* = 0.034) and 26.5% (*p* = 0.022), respectively. Spatial changes in historical carbon stocks are strongly associated with land use changes that occurred between 1990 and 2018. We estimated a non-significant loss of 61.9 Tg-C (*p* = 0.726) from the total carbon stocks (calculated as the sum of AGB, BGB, and SCS) of which 80% of the losses came from soil stocks, 11% from BGB, and 8% from AGB. These findings reveal the need to monitor carbon stocks in sandy soils to reduce the uncertainties of estimates and support the development of effective sustainable agriculture policies. The best alternatives for reducing carbon losses in the Cerrado are to maintain natural forest cover and to recover soils through sustainable soil management, especially in pasturelands where soil carbon stocks are lowest.

## Introduction

Land use and land use changes together comprise the second-largest source of carbon (C) emissions, representing 23% of total anthropogenic greenhouse gas emissions between 2007 and 2016 [1]. These emissions result from altering the balance between inflows and outflows of carbon reservoirs due to the removal of natural vegetation for agricultural practices [2–6].

Among terrestrial carbon reservoirs, soil is the largest global pool of stored carbon (1500 Pg-C in the first meter of soil), holding two to four times more carbon than the combined aboveground and belowground biomass pool, estimates of which range from 385 to 650 Pg-C [7,8]. In addition to the fact that soils have the highest carbon storage in the terrestrial system, they are also crucial for maintaining carbon cycling in the biosphere and for ensuring global food security [9,10]. Soils are a key element for achieving the Sustainable Development Goals proposed by the United Nations Conference on Environment and Development, since wise soil management can facilitate the provisioning of full ecosystem services; these include supporting, maintaining, or improving soil fertility and productivity as well as increasing ecosystem resilience in a changing climate [8,11,12]. Different soil management practices can result in enhancement or depletion of carbon uptake, leading soils to be carbon sinks [13–17] or sources to the atmosphere [18–22]. Practices like no-tillage, crop rotation, and integrated crop-livestock systems have been recommended by the Intergovernmental Panel on Climate Change to reduce the carbon emissions associated with land use change and reach the targets proposed by the Paris Agreement [1].

In Brazil, the largest source of carbon emissions due to land use change is native vegetation suppression [23,24]. Although deforestation rates in Brazil’s Amazonia region are notoriously high, they are lower than the rates associated with suppression of Cerrado native vegetation: in 2015, this rate in the Cerrado region was 52% higher than the rate of deforestation in the Amazon [25]. The vegetation of the Cerrado biome is a mosaic of different vegetation types ranging from grasslands to forestlands, and it has been classified into three categories by [26]: forestlands (ciliary forest, gallery forest, dry forest, and *Cerradão*), shrublands (*Cerrado sensu stricto*, park savanna, palm, and *vereda*), and grasslands (*campo limpo*, *campo sujo*, and *campo rupestre*). Cerrado soils contain about 34% of Brazilian soil carbon stocks (71.3 Pg-C), storing 24 Pg-C within the top meter [27,28].

Until the 1970s, Cerrado lands in Brazil remained unused for agriculture since they were considered unproductive, mainly due to their acidic and nutrient-poor soils [29]. This situation changed in the 1970s due to depletion of the available lands for agricultural occupation in the Brazilian South and Southeast [30,31] and due to technological advances in soil management and the use of specific techniques, such as liming (correction of acidity through application of limestone) and fertilization with phosphate and potash.

The need to increase Brazilian agricultural production and offers of government incentives for regional development programs led farmers to migrate from Southern Brazil to Cerrado lands in the Brazilian Center-West (mainly to the states of Goiás, Mato Grosso, and Mato Grosso do Sul) [32]. In these areas, the seasonal climate, with average annual precipitation between 1,000 and 1,500 mm, in combination with good physical soil conditions such as fine texture, high permeability, and great depth, provided favorable conditions for agricultural expansion, despite the naturally acidic and nutrient-poor soils.

After the land occupation of the Brazilian Center-West, the agricultural frontier expanded to the borders of the Cerrado biome, where soils are predominantly sandy and interannual climate variability is higher, giving birth to the newest agricultural frontier of the country: MATOPIBA a region comprising parts of the states of Maranhão (MA), Tocantins (TO), Piauí (PI), and Bahia (BA) [33]. In this region, a particular area known as Western Bahia, stands out for being responsible for 50% of all grain produced in MATOPIBA.

Western Bahia has an abundance of natural resources (especially water availability) and a flat topography, which facilitated the development of agriculture in the region [34,35]. The cropland area in this region expanded from virtually zero in 1985 to 690 thousand hectares of soybeans in 2000 to 1.6 million hectares in 2018, reaching production records of 6 million tons of soybeans and 1.245 million tons of cotton produced in the 2017/2018 harvest [36]. This rapid advance of agribusiness in Western Bahia exposed the natural resources to intensive anthropic pressure, raising concerns about the effects of land use change on regional water availability, landscape fragmentation, soil physical properties, and carbon storage [16,37–40]. Previous studies have shown that suppression of native vegetation throughout the Cerrado domain caused nearly 200 Tg-C to be emitted between 2003 and 2008 (40 Tg-C yr^−1^) from areas converted to pasture [41] and an average of 179 Tg-C between 2003 and 2013 from areas converted to cropland (18 Tg-C yr^−1^) [42]. However, these studies considered average carbon stock estimates based on field data collected mainly in clayey soils and under different vegetation physiognomies throughout the Cerrado domain, providing only a limited perspective on actual carbon dynamics from land use change in this biome. For MATOPIBA, especially in the Western Bahia region, where the soils are predominantly sandy, there is a lack of knowledge of the actual amounts and spatial patterns of carbon stored in the aboveground biomass (AGB), belowground biomass (BGB), and soil reservoirs, hindering determination of accurate estimates of carbon balance for agricultural areas and decisions regarding the best management strategies.

In this context, and considering the relevance of knowledge about carbon stocks for achieving the Sustainable Development Goals, it is extremely relevant to quantify the sources and sinks of carbon on the largest and most dynamic agricultural frontier in Brazil to provide a basis for the development of climate change mitigation and food security policies. Thus, this study reports soil carbon stocks (SCS) for three agricultural land uses (LUs) and two native vegetation land covers (LCs), and it develops a historical reconstruction of carbon stocks for 1990–2018 to evaluate how land use changes are affecting carbon dynamics in the agricultural frontier of the Cerrado.

## Methods

### Study area

**Fig 1:**
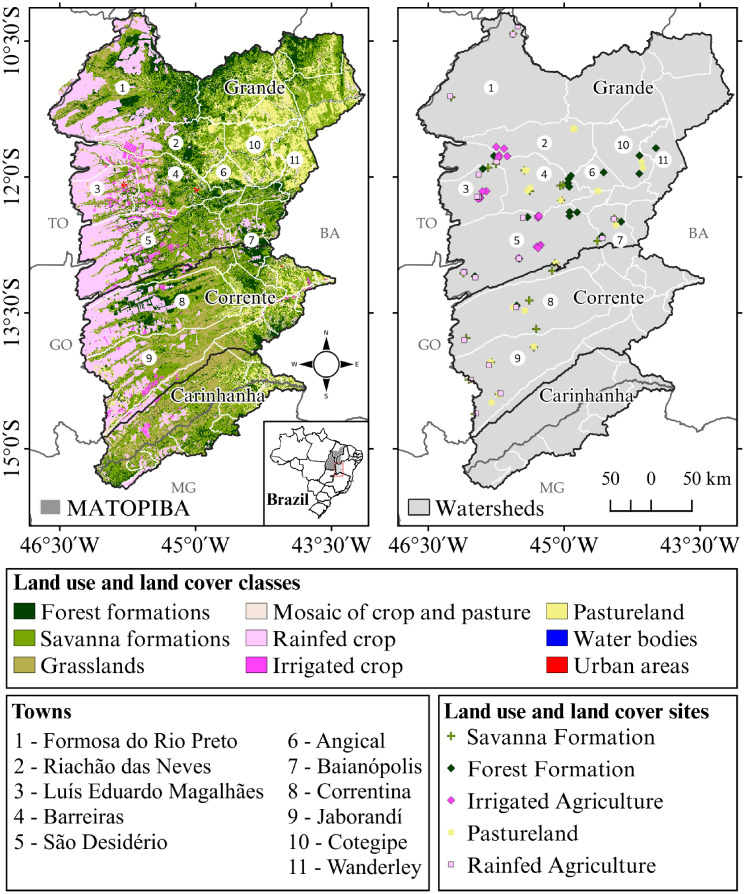
Study area and locations of the soil sample collection sites and main towns in the region. Symbol colors represent different land use and land cover classes. This figure was made using open source geographic information system (QGIS) and open data source: watersheds vector lines and the land use and land cover classification map were obtained from OBahia (http://obahia.dea.ufv.br), and geopolitical borders were obtained from IBGE (https://mapas.ibge.gov.br).

The study area, Western Bahia, is located on top of the Urucuia aquifer and is drained by three important river basins (the Rio Grande, Rio Corrente, and Rio Carinhanha), which have a combined area of 131,168 km^2^ (Fig 1). This aquifer plays a key role in Western Bahia’s agricultural activities, since it is responsible for water flow regulation during the dry season [43], allowing the use of irrigation throughout most of the year.

The soils of Western Bahia have developed on the geological formation of the Urucuia Group (Upper Cretaceous), which is dominated by quartz sandstone cemented by siliceous materials. The soils are sandy (clay content below 40%, with sand fraction between 70% and 90%) [40], deep, well drained, acidic (with high aluminum and Fe-oxide content), and of low fertility [44]. According to the Brazilian Soil Classification System, the predominant soils classes are Latossolos and Neossolos (Fig 1), which are strongly associated with the typical vegetation of the Cerrado [34,45]. The Cerrado *sensu stricto* is the main physiognomy in this region, with low stocks of dry AGB ranging from 9.2 to 12.6 Mg ha^-1^ [46].

The natural landscape is characterized as flat or mildly hilly, which allows for the use of intensive mechanization and of chemical inputs for correction of fertility, providing favorable conditions for both agricultural extensification and intensification. The altitude gradually decreases from a height of 1,083 m in the extreme west, where the formations known as “*chapadões*” (tablelands) dominate, to between 880 m and 680 m in the Central region, and then to between 580 m and 380 m at the eastern border.

The regional climate is tropical humid (Aw according to the Köppen climate classification) [47] and has two well-defined seasons: dry (April to September) and rainy (October to March). The average annual rainfall ranges from ~700 mm yr^−1^ in the east to > 1,400 mm yr^−1^ in the extreme west [38].

### Soil sampling design (field work)

We collected soil samples in two field campaigns in 2017 for two native vegetation LCs and three agricultural LUs (Fig 1). These land use and land cover classes (LULCCs) are (1) Cerrado (CDO), including both savanna and grassland formations; (2) forest formations (FOR); (3) rainfed agriculture (RAG); (4) irrigated cropland (IRR); and (5) pasturelands (PAST). The CDO class is mainly represented by *Cerrado sensu stricto* and grassland areas with no signs of human intervention such as fire, erosion, or deforestation. The FOR class is represented by samples collected in forest and *Cerradão* areas. The RAG and IRR areas are used to cultivate crops (soybean, maize, cotton, and bean) and were sampled in different stages of the crop cycle. The IRR samples were collected in the middle of the crop cycle, during July of 2017, while RAG samples were collected during November and December of 2017, at the beginning of the rainfed-crop cycle. The CDO, FOR, and PAST soil samples were also collected during November and December of 2017. The PAST samples were collected in both well-managed pastures (areas under pasture rotation, without overgrazing) and degraded pastures with poor management (areas with termite mounds, soil compaction, and low grass cover).

Each LULCC was sampled at 20 sampling points following mainly logistical criteria, including road conditions and receipt of permission to enter the farm. It was not necessary to request authorization from federal or state agencies to carry out this experiment. All samples were collected from private properties (farms) and their owners requested confidentiality regarding the information on the properties. Some CDO and FOR areas were sampled along the road between farms in natural vegetation areas. All sampled geographic coordinates are available in S1 Table. In total, 1,400 soil samples (700 undisturbed and 700 disturbed) were collected (5 LULCCs × 20 sampling points × 7 depth levels). At each sampling point, samples were collected at the depths 0–5, 5–10, 10–15, 15–20, 20–40, 40–60, and 60–100 cm. The disturbed samples were composed of three subsamples collected and homogenized in the field. The three deeper soil layers were considered homogeneous, and samples were collected in the middle of the layer at 30, 50, and 70 cm, respectively.

We used the 700 undisturbed samples to calculate the soil bulk density (g cm^−3^) and the 700 disturbed samples to estimate soil organic carbon concentration (g kg^−1^), using the ring method [48] and the colorimetric Walkley–Black method [49], respectively. These calculated properties were used with the equivalent mass method of [50] to calculate the SCS for the layers SCS_30_ (0–30 cm in depth), SCS_60_ (0–60 cm), and SCS_100_ (0–100 cm). This method uses a particular mass of reference soil (from natural land cover) to remove possible effects of soil compaction in the carbon stock estimates, especially in the agricultural soils.

Student´s t-test was used to compare the differences in SCS_30_ and SCS_100_ between LULCCs. Descriptive statistics (mean, standard deviation, and confidence interval) were also calculated.

### Land use and land cover databases for historical carbon reconstruction

The land use and land cover classification map dataset OBahia (available at http://obahia.dea.ufv.br/maps) was used for the historical reconstruction of the AGB, BGB, and SCS_100_ spatial patterns. The OBahia classification has nine LULCCs at a scale of 1:15,000 (30 m spatial resolution): forest formations (FOR), savanna formations (SF), grassland formations (GF), mosaic of agriculture and pasturelands (RAG/PAST), rainfed agriculture (RAG), irrigated agriculture (IRR), pasturelands (PAST), water bodies, and urban areas/farm buildings. This database was developed by filtering the Landsat 5, 7, and 8 satellite image collections for the dry season (04 April to 30 September) for each year from 1990 to 2018, and it was classified through the Random Forest classifier in the Google Earth Engine platform. The results of the OBahia classification show trends of agricultural use in the region over time with an accuracy of 90% [51]. Although the total spatial and temporal variability of LULCCs are not the focus of this study, a summary is presented in Table 1 to provide a more cohesive discussion about the relationship between estimated carbon stocks and the landscape dynamics. The 2018 spatial pattern of LULCCs is also presented in Fig 1a, identifying the regions where the soil samples for each LULCC were collected.

**Table 1:** Distribution of land use and land cover areas in Western Bahia in 1990, 1997, 2004, 2011, and 2018.

Land use and land cover classes	Area (Mha)				
	1990	1997	2004	2011	2018
Forest formations – FOR	3.34	3.29	3.06	2.81	2.07
Savanna formations – SF	4.27	4.24	4.07	3.77	4.49
Grassland formations – GF	3.77	3.36	3.43	3.14	1.69
Mosaic of RAG and PAST – RAG/PAST	0.08	0.06	0.05	0.05	0.36
Rainfed agriculture – RAG	0.84	1.15	1.43	2.14	3.08
Irrigated cropland – IRR	0.02	0.05	0.06	0.08	0.20
Pasture – PAST	1.08	1.24	1.30	1.39	1.51
Natural vegetation	11.39	10.89	10.56	9.72	8.25
Agricultural lands	2.02	2.51	2.84	3.67	5.14
Total area	13.40	13.40	13.40	13.40	13.40

The nine OBahia classes are associated with the five LULCCs used in fieldwork for the SCS_100_ historical reconstruction. In this study, natural land cover is represented by forest formations (FOR) and Cerrado formations (CDO), with the latter subdivided into savanna formations (SF) and grassland formations (GF). The vegetation cover in the SF class is predominantly shrubs and trees, while grasses are predominant in the GF vegetation cover class. Here, we use different values for AGB and BGB for SF and GF, although we use a single set of values for soil carbon for the Cerrado, which was collected from both SF and GF areas. The agricultural LUs are divided into rainfed agriculture (RAG), irrigated agriculture (IRR), pasture (PAST), and mosaic of agriculture and pasture (RAG/PAST).

### Spatial reconstruction of historical carbon stocks (1990–2018)

Most global AGB datasets from remote sensing depend on parameters related to forest biomass such as height, leaf area index, or net primary production. The scarcity of field data and the lack of a model able to represent savannas in tropical regions across the world has led to underestimation of AGB in woodlands and overestimation in grasslands and savannas [52]. In tropical woody savannas, where the vegetation is strongly controlled by climate seasonality and there are a large number of deciduous species, AGB estimates from remote sensing show high uncertainties [52,53] and, for the most part, are not consistent between tropical regions of Africa and South America [54]. Thus, we did not use remote sensing estimates of AGB, and instead we assigned AGB and BGB values to each LULCC using values from the literature (Table 2). When available, we chose studies that estimated the average and standard deviation for AGB and BGB in Western Bahia, and when not available, we used studies from nearby Cerrado areas. For BGB estimates in RAG and IRR, we used a proxy based on [55], who reports that the ratio BGB/AGB is about 45% for irrigated soybean crops and 70% for rainfed soybean crops.

**Table 2:** Parameters for Land Use and Land Cover Classes (LULCCs) used in spatial reconstruction of historical carbon stocks: Forest formations (FOR), savanna formations (SF), Grassland Formations (GF), cropland or pastureland (RAG/PAST), rainfed cropland (RAG), irrigated cropland (IRR), and pastureland (PAST).

Aboveground Biomass in Mg-C ha^−1^					
**LULCC**	Avg	Std	Min*	Max*	Reference for Avg and Std values
FOR	12.06	0.12	11.83	12.29	Santana et al. (2013)^1^
SF	6.771	1.135	5.693	7.849	Oliveira et al. (2019)^1^
GF	3.468	2.018	1.551	5.385	Miranda et al. (2014)^2^
RAG	6.010	1.194	4.876	7.144	Cruz et al. (2010)^1^
IRR	7.428	2.348	5.197	9.659	Silva et al. (2018) ^2^
PAST	1.315	0.929	5.198	6.118	Santos et al. (2007)^2^
RAG/PAST	3.663	1.062	5.197	8.679	Average of RAG and PAST
Belowground Biomass in Mg-C ha^-1^					
FOR	8.638	1.633	7.087	10.19	Miranda et al. (2014)^2^
SF	16.27	6.474	10.12	22.42	Miranda et al. (2014)^2^
GF	8.109	4.849	3.502	12.72	Miranda et al. (2014)^2^
RAG	4.207	0.836	3.413	5.001	Zilio (2014)
IRR	3.343	1.057	2.329	4.357	Zilio (2014)
PAST	2.622	1.285	1.401	3.843	Santos et al. (2007)^2^
RAG/PAST	3.414	1.060	2.407	4.421	Average of RAG and PAST
Soil Carbon Stock (0–100 cm) in Mg-C ha^-1^					
FOR	82.50	43.60	41.08	123.9	Estimated from fieldwork data (Table 3)
SF and GF (CDO)	70.30	41.60	30.78	109.8	
RAG	57.40	33.30	25.77	89.03	
IRR	78.10	25.50	53.88	102.3	
PAST	51.70	25.50	27.48	75.92	
RAG/PAST	62.40	28.10	35.71	89.09	

^1^ Studies that measured aboveground biomass in Western Bahia.

^2^ Studies that measured aboveground biomass or belowground biomass in the Cerrado domain.

*Maximum and minimum values estimated using Monte Carlo simulation, assuming a normal distribution with *n* = 10^5^, and the top and bottom 2.5% percentiles.

**Fig 2:**
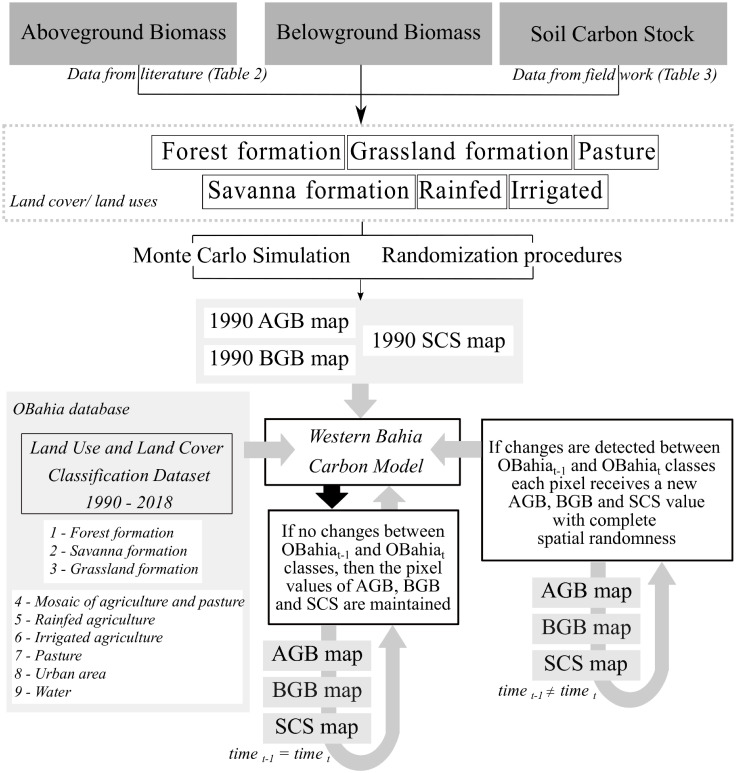
Flowchart for the computation of historical carbon stocks reconstruction using the WBCM.

The average and standard deviation of AGB and BGB described in the literature and the SCS at 100 cm depth (SCS_100_) obtained by fieldwork were used to estimate LULCC population sizes (*n* = 7 for AGB and BGB; *n* = 6 for SCS_100_) by assuming a normal distribution and the number of pixels for each population in 1990. These LULCC populations were used to compose a database in which maximum and minimum values were set to the top and bottom 2.5% percentiles, respectively. From these databases, we built the initial (1990) maps of AGB, BGB, and SCS_100_. Each pixel (30 m) of the initial biomass map received a random AGB and BGB value according to the corresponding class determined by the OBahia classification (Fig 2).

This method introduces uncertainties in the AGB, BGB, and SCS_100_ estimates, because it does not consider the effects of altitude, soil types, or fertility on the biomass spatial distribution. AGB and BGB values were randomly chosen for all pixels within the same LULCC. For SCS_100_, the uncertainties are part of the results of this study and are presented in Table 3.

**Table 3:** Average soil carbon stocks in Mg-C ha^−1^ for depths of 0–30 cm, 0–60 cm, and 0–100 cm for different land use and land cover classes in Western Bahia.

		0–30 cm			0–60 cm			0–100 cm		
LULCCs	*n*	*avg*	*std*	*ci*	*avg*	*std*	*ci*	*avg*	*std*	*ci*
FOR	19	51.0	25.9	12.5	63.4	33.2	16.0	82.5	43.6	21.0
IRR	20	45.5	11.4	5.18	61.2	15.1	6.91	78.1	25.5	11.8
CDO	23	40.1	23.3	10.1	51.4	29.2	12.6	70.3	41.6	18.0
RAG	20	32.3	20.4	9.6	42.6	26.6	12.5	57.4	33.3	15.6
PAST	21	28.0	11.4	11.4	36.8	15.1	15.7	51.7	25.5	19.7

Averages (*avg*) followed by standard deviations (*std*) and confidence intervals (*ci*) at α = 0.05.

The carbon stock historical reconstruction was calculated using a simple bookkeeping model, the Western Bahia Carbon Model (WBCM). This model tracks changes in carbon stocks from year to year rather than modeling individual biological processes that constitute the carbon balance. The lack of knowledge about long-term carbon annual gains or losses to the AGB, BGB, and SCS_100_ for each LULCC limited the use of growth and mortality rates in the carbon reconstruction maps. Thus, this model assumes that (1) AGB, BGB, and SCS_100_ for the CDO and FOR classes are at equilibrium for all LULCCs; (2) no changes occurred in the last 28 years in the SCS for natural LCs; (3) the croplands in IRR and RAG areas are annual croplands, with the same AGB/BGB ratio; and (4) there are no interactions between the carbon reservoirs.

The first step of the historical carbon reconstruction is to provide the AGB, BGB and SCS_100_ maps for the first year of the period (1990) and the land cover classification dataset from OBahia (1990–2018) as inputs. The model follows the same procedure to generate a new map for the subsequent years. First, if there were no changes in classes between OBahia_t−1_ and OBahia_t_, then the values of AGB, BGB, and SCS_100_ are maintained. Otherwise, if changes are detected, each pixel receives new AGB, BGB, and SCS_100_ values according to the database estimated from Table 2. Finally, at the end of each year, the WBCM writes the map of AGB, BGB, and SCS_100_ in Mg-C ha^-1^ (Fig 2).

We used the spatial patterns of AGB, BGB, SCS_100_, and total carbon stocks (TCS) to compare the significance of differences in the carbon reservoirs using Student´s t-test for four periods: 1990–1996, 1997–2003, 2004–2010, and 2011–2018. TCS represents the sum of the AGB, BGB, and SCS_100_ reservoirs. The rates of change of AGB, BGB, SCS_100_, and TCS for each period and the differences in TCS between the first years of each period are also presented.

## Results

### Soil carbon stocks in Western Bahia—Observed data

The average, standard deviation, and confidence interval for SCS_30_, SCS_60_, and SCS_100_ values for each LULCC are presented in Table 3. The SCS_30_ and SCS_100_ differences between agricultural LUs and native vegetation covers are presented in Table 4, while *p*-values for Student’s t-test are presented in Table 5.

**Table 4:** Percentage differences in soil carbon stocks between agricultural LUs and native vegetation LCs in Western Bahia for 0–30 cm and 0–100 cm depths.

	0–30 cm		0–100 cm	
LULCC	FOR	CDO	FOR	CDO
IRR	−12.2%	11.8%	−5.34%	11.1%
RAG	−57.7%	−23.9%	−30.3%	−18.2%
PAST	−82.1%	−43.1%	−37.3%	−26.4%

**Table 5:** *p*-values obtained by Student’s t-test with α = 0.05 for differences in the means between soil carbon values of agricultural LUs and native vegetation LCs in Western Bahia for 0–30 cm and 0–100 cm depths.

*0***–***30 cm layer*					
	FOR	IRR	CDO	RAG	PAST
FOR	-	0.399	0.164	0.018	0.007
IRR	0.399	-	0.332	0.018	0.007
CDO	0.164	0.332	-	0.254	0.107
RAG	0.018	0.018	0.254	-	0.547
PAST	0.007	0.007	0.107	0.547	-
*0***–***100 cm layer*					
	FOR	IRR	CDO	RAG	PAST
FOR	-	0.704	0.363	0.053	0.031
IRR	0.704	-	0.455	0.034	0.022
CDO	0.363	0.455	-	0.269	0.155
RAG	0.053	0.034	0.269	-	0.635
PAST	0.031	0.022	0.155	0.635	-

FOR, IRR, and CDO areas have higher SCS_30_ compared with other LULCCs, with 51.0 ± 25.9 Mg-C ha^−1^, 45.5 ± 11.4 Mg-C ha^−1^ and 40.1 ± 23.3 Mg-C ha^−1^ (average followed by standard deviation), respectively (Table 3). In RAG and PAST, SCS_30_ values are 32.3 ± 20.4 Mg-C ha^−1^ and 28.0 ± 11.4 Mg-C ha^−1^, respectively. These results reveal that the highest SCS losses in the top soil layer (0− 30 cm) may happen when FOR or CDO areas are replaced with PAST, since in our study these changes reduced the SCS_30_ by 82.1% and 43.1%, respectively (Table 4). The former changes are statistically significant at *p* = 0.007, while the latter are not (*p* = 0.107) (Table 5).

The replacement of FOR with RAG decreased SCS_30_ by 57.7% (*p* = 0.018). Other conversions of native vegetation (CDO to RAG, FOR to IRR, and CDO to IRR) did not significantly change SCS_30_ (CDO to RAG, *p* = 0.254; FOR to IRR, *p* = 0.399; CDO to IRR, *p* = 0.332). On the other hand, the use of IRR practices increased SCS_30_ by 41% when compared with RAG (*p* = 0.018) and by 62.5% when compared with PAST (*p* = 0.007) (Table 5).

The patterns for SCS_60_ are similar to those found for SCS_30_. The average SCS_60_ values for FOR, IRR, and CDO areas were 63.4 ± 33.2 Mg-C ha^−1^, 61.2 ± 15.1 Mg-C ha^−1^, and 51.4 ± 29.2 Mg-C ha^−1^, respectively (Table 3). For RAG and PAST, the SCS_60_ values were 42.6 ± 26.6 Mg-C ha^−1^ and 36.8 ± 15.1 Mg-C ha^−1^, respectively (Table 3).

Considering the full soil profile (0–100 cm), SCS_100_ estimates for FOR and CDO were 82.5 ± 43.6 Mg-C ha^−1^ and 70.3 ± 41.6 Mg-C ha^−1^, respectively (Table 3). For the agricultural LUs, the SCS_100_ was 78.1 ± 25.5 Mg-C ha^−1^ for IRR, 57.4 ± 33.3 Mg-C ha^−1^ for RAG, and 51.7 ± 25.5 Mg-C ha^−1^ for PAST. As with SCS_30_, these results show that higher SCS_100_ losses occurred when FOR or CDO areas were replaced with PAST, reducing the SCS_100_ by 37.3% (*p* = 0.031) and 26.4%, respectively (*p* = 0.155) (Tables 4 and 5).

The replacement of FOR and CDO with RAG decreased SCS_100_ by 30.3% (*p* = 0.053) and 18.2% (*p* = 0.269), respectively. Conversion of FOR to PAST decreased SCS_100_ by 37.3% (*p* = 0.031). Other conversions of native vegetation did not significantly change SCS_100_ (FOR to IRR, *p* = 0.704; CDO to IRR, *p* = 0.455; CDO to PAST, *p* = 0.155) (Tables 4 and 5). The replacement of PAST with IRR significantly increased SCS_100_ by 34% (*p* = 0.022), while replacement of RAG with IRR led to a significant increase of 26.5% (*p* = 0.034) (Table 5).

In summary, for both the 0–30 cm and 0–100 cm depths, conversion of forestlands to pasturelands and rainfed agriculture is very likely to decrease soil C stocks (*p* < 0.05), while the introduction of irrigation practices in rainfed croplands or pasturelands is very likely to increase soil C stocks (*p* < 0.05). All other combinations of LC or LU conversions yield non-significant changes in soil C.

### Spatial and temporal patterns of carbon stocks in Western Bahia

The main AGB losses in Western Bahia occurred due to accelerated clearing of natural vegetation and replacement with agricultural land use managed with different practices, such as RAG, PAST, and IRR. The patterns of changes in LULCCs show that in 2018 compared with 1990, FOR and GF areas decreased from 3.34 Mha to 2.07 Mha (38.1%) and from 3.77 Mha to 1.69 Mha (55%), respectively, while SF increased by 0.22 Mha (5.1%) (Table 1). Between 1990 and 2018, the agricultural expansion in Western Bahia increased RAG areas by 264% (from 0.84 Mha to 3.08 Mha), PAST areas by 40% (from 1.08 Mha to 1.51 Mha), and IRR areas by 1000% (from 0.02 Mha to 0.20 Mha) (Table 1). Our estimates indicate that AGB carbon storage for agricultural classes (RAG + IRR + PAST + RAG/PAST) increased from 11.5 ± 2.42 Tg-C in 1990 to 30.2 ± 2.39 Tg-C in 2018 (Table 6). However, these increases in AGB were not enough to compensate for AGB losses from natural areas, which decreased from 82.3 ± 1.69 Tg-C to 61.2 ± 1.69 Tg-C over the same period (Table 6).

**Table 6:** Aboveground biomass, belowground biomass, soil C stocks and total C stocks for natural land covers (FOR, SF, GF) and agricultural land uses (RAG, IRR, PAST, RAG/PAST) in Western Bahia.

LULCC	Aboveground Biomass - AGB									
	1990		1997		2004		2011		2018	
	Tg-C		Tg-C		Tg-C		Tg-C		Tg-C	
	Avg	Std	Avg	Std	Avg	Std	Avg	Std	Avg	Std
FOR	40.3	0.059	36.9	0.06	33.8	0.061	27.5	0.061	24.9	0.063
SF	28.9	0.586	27.6	0.586	25.5	0.585	29.3	0.585	30.4	0.585
GF	13.1	1.04	11.9	1.04	10.9	1.04	8.09	1.04	5.88	1.04
RAG/PAST	0.445	0.416	0.302	0.420	0.311	0.424	0.564	0.427	2.04	0.429
RAG	5.07	0.617	8.61	0.616	12.9	0.616	16.9	0.616	18.5	0.616
IRR	0.132	1.21	0.437	1.21	0.616	1.21	0.80	1.21	1.45	1.21
PAST	5.84	0.178	7.04	0.158	7.59	0.146	7.79	0.141	8.27	0.130
Natural	82.3	1.69	76.3	1.69	70.3	1.69	64.9	1.69	61.2	1.69
Agricultural	11.5	2.42	16.4	2.41	21.4	2.40	26.1	2.40	30.2	2.39
Total AGB	93.7	4.11	92.7	4.09	91.7	4.09	91.0	4.08	91.4	4.08
Belowground Biomass - BGB										
FOR	28.9	0.843	26.4	0.843	24.2	0.843	19.7	0.843	17.9	0.843
SF	69.5	3.34	66.3	3.34	61.3	3.34	70.3	3.34	73.0	3.34
GF	30.6	2.50	27.8	2.50	25.5	2.50	18.9	2.50	13.7	2.50
RAG/PAST	0.270	0.548	0.182	0.549	0.187	0.549	0.339	0.549	1.23	0.549
RAG	3.55	0.432	6.03	0.432	9.02	0.432	11.8	0.432	12.9	0.432
IRR	0.06	0.549	0.197	0.550	0.277	0.550	0.360	0.551	0.653	0.551
PAST	2.83	0.663	3.40	0.663	3.65	0.663	3.75	0.662	3.97	0.662
Natural	129	6.68	120	6.68	111.1	6.68	109.0	6.68	104.6	6.68
Agricultural	6.71	2.19	9.81	2.19	13.1	2.19	16.3	2.19	18.8	2.19
Total BGB	136	8.88	130	8.88	124	8.88	125	8.88	123	8.88
Soil Carbon Stock - SCS_100_										
FOR	276	22.5	252	22.5	231	22.5	188	22.5	171	22.5
SF	300	21.5	286	21.5	265	21.5	304	21.5	315	21.5
GF	265	21.5	241	21.5	221	21.5	164	21.5	119	21.5
RAG/PAST	4.89	14.5	3.32	14.5	3.42	14.5	6.19	14.5	22.4	14.5
RAG	48.4	17.2	82.3	17.2	123	17.2	162	17.2	177	17.2
IRR	1.39	13.2	4.60	13.2	6.48	13.2	8.41	13.2	15.3	13.2
PAST	55.9	13.2	67.0	13.2	72.1	13.2	73.9	13.2	78.3	13.2
Natural	841	65.4	779	65.4	717.	65.4	656	65.4	605	65.4
Agricultural	111	58.0	157	58.0	205	58.0	250	58.0	292	58.0
Total SCS_100_	951	123	936	123	922	123	906	123	897	123
TCS	1,18	136	1,16	136	1,14	136	1,12	136	1,11	136

The total BGB decreased by 9.9% between 1990 and 2018, reducing the BGB storage from 136 ± 8.88 Tg-C to 123 ± 8.88 Tg-C (Table 6). This reduction is a result of changes in BGB reservoirs under natural areas that decreased from 129 ± 6.7 Tg-C to 105 ± 6.7 Tg-C and increased in agricultural areas from 6.71 ± 2.2 Tg-C to 18.8 ± 2.2 Tg-C (Table 6).

The sizes of SCS_100_ varied across LULCCs and over time. In 1990, the SCS_100_ in natural vegetation areas totaled 841 ± 65.4 Tg-C (88.4% of the total across all LULCCs), while agricultural LU stocks were 111 ± 58 Tg-C (12% of the total). Across the agricultural land use classes, SCS_100_ storage was distributed as follows: 51% of the stocks were in PAST (55.9 ± 13.2 Tg-C), followed by RAG with 44% (48.4 ± 17.2 Tg-C), RAG/PAST with 4.42% (4.89 ± 14.5 Tg-C), and IRR with 1.25% (1.39 ± 13.2 Tg-C).

In 2018, the SCS_100_ in natural vegetation decreased to 604.8 ± 65.4 Tg-C (67.4% of the total), while storage in agricultural LU classes reached 292.4 ± 58 Tg-C (32.6% of the total) (Table 6). Of the total SCS_100_ in 2018, 60.4% of the stocks were in RAG (177 ± 17.2 Tg-C), followed by PAST with 26.8% (78.3 Tg-C ± 13.2), RAG/PAST with 7.66% (22.4 ± 14.5 Tg-C), and IRR with 5.22% (15.3 ± 13.2 Tg-C).

The increases of carbon stocks in agricultural land use classes from 1990 to 2018 summed to an overall increase of 212.7 Tg-C (from 11.5 Tg-C to 30.2 Tg-C for AGB, 6.71 Tg-C to 18.8 Tg-C for BGB, and 111 Tg-C to 292.4 Tg-C for SCS_100_). However, this was not enough to compensate for carbon losses from natural areas, which totaled 281.1 Tg-C. In 1990, the SCS_100_ in native vegetation represented 88.4% (841 ± 65.4 Tg-C) of TCS in Western Bahia, while in 2018, the SCS_100_ in native vegetation represented only 67.4% (604.8 ± 65.4 Tg-C) (Table 6).

Although our results identify carbon losses in all carbon reservoirs, with soils as the major contributor, the changes are non-significant (Tables 7 and 8). Between the periods 1990–1996 and 2011–2018, AGB, BGB, and SCS_100_ decreased by 3.94% (*p* = 0.858), 7.02% (*p* = 0.809), and 5.17% (*p* = 0.758), respectively. This decreased the average of the total carbon stock from 87.5 Mg- C ha^-1^ to 82.9 Mg- C ha^-1^ (Table 7). Considering that Western Bahia covers 13.4 Mha (Table 1), we estimate that the total carbon stock for all of Western Bahia decreased from 1,172 Tg-C in the period 1990–1996 to 1,110 Tg-C in 2011–2018, corresponding to an emission of 61.9 Tg-C (a reduction of 5.28%; *p* = 0.726) to the atmosphere (Table 8).

**Table 7:** Averages and standard deviations of annual values for aboveground biomass (AGB), belowground biomass (BGB), and soil carbon stocks (SCS_100_) in Mg-C ha^−1^, and their relative percent contributions to total carbon stocks (TCS) for the periods 1990–1996 (*n = 7*), 1997–2003 (*n = 7*), 2004–2010 (*n = 7*), and 2011–2018 (*n = 8*) in Western Bahia.

	1990–1996 (P1)				1997–2003 (P2)				2004–2010 (P3)				2011–2018 (P4)			
	%	avg	std	n	%	avg	std	n	%	avg	std	n	%	avg	std	n
AGB	8.01	7.01	3.21	7	8.00	6.86	3.09	7	8.10	6.85	2.95	7	8.12	6.73	2.54	8
BGB	11.4	9.97	5.21	7	11.1	9.51	5.28	7	10.9	9.22	5.38	7	11.2	9.27	5.80	8
SCS	80.6	70.5	22.7	7	80.9	69.4	22.6	7	81.0	68.5	22.4	7	80.7	66.9	21.9	8
TCS	100	87.5	24.9	7	100	85.7	25.0	7	100	84.5	25.0	7	100	82.9	24.8	8

**Table 8:** Percentage changes in carbon reservoir means for the periods 1997–2003 (P2), 2004–2010 (P3), and 2011–2018 (P4) compared with the period 1990–1996 (P1) in Western Bahia.

	P2 − P1			P3 − P1			P4 − P1		
	%	df	*p*-value	%	df	*p*-value	%	df	*p*-value
AGB	−2.08	12	0.933	−2.31	12	0.923	−3.94	13	0.858
BGB	−4.57	12	0.874	−7.52	12	0.796	−7.02	13	0.809
SCS	−1.63	12	0.926	−2.95	12	0.866	−5.17	13	0.758
TCS	−2.00	12	0.898	−3.42	12	0.827	−5.28	13	0.726

Percentages (%) followed by degrees of freedom (df) and their significance according to Student’s t-test (p-value) at α = 0.05.

**Fig 3:**
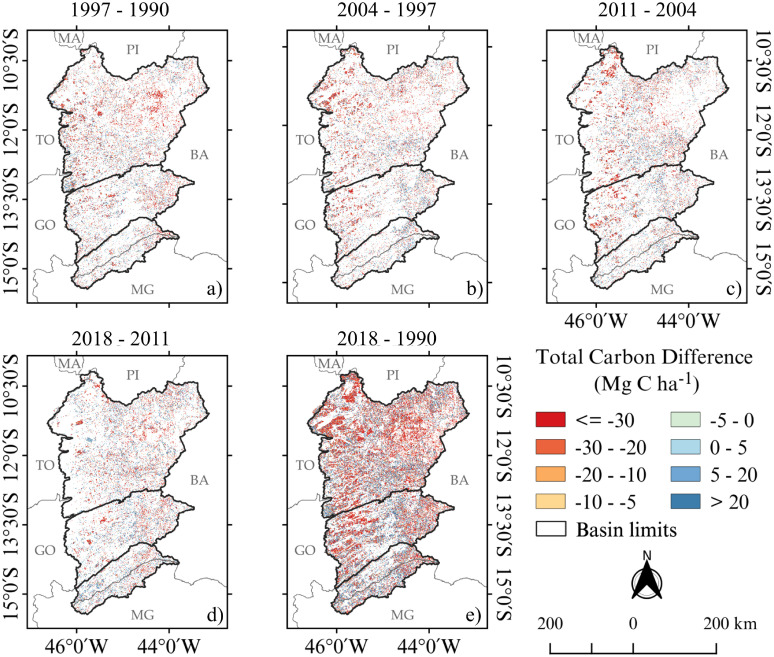
Differences in total carbon stocks for Western Bahia. Carbon stocks in Mg-C ha^−1^ for (a) 1997 − 1990, (b) 2004 − 1997, (c) 2011 − 2004, (d) 2018 − 2011, and (e) 2018 − 1990. This figure was made using open source geographic information system (QGIS) and open data source: watersheds vector lines were obtained from OBahia (http://obahia.dea.ufv.br) and geopolitical borders were obtained from IBGE (https://mapas.ibge.gov.br).

**Fig 4:**
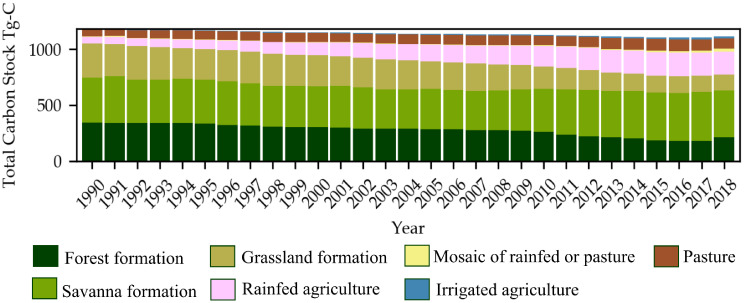
Total carbon stock temporal variability for different LULCCs for Western Bahia, 1990–2018.

Over the years of the study period, total C losses increased (Fig 3). Spatially, these losses, occurring between 1990 and 2018, were found mainly in the Rio Grande and Rio Corrente basins. Losses above 30 Mg-C ha^−1^ were found mainly in the northwest of Western Bahia, near the border with Tocantins and Piauí, where the agricultural practices are older compared with agriculture in the Corrente and Carinhanha basins (Fig 3). Historically, TCS in Western Bahia progressively declined over the years from 1,172 Tg-C in 1994 to 1,138 Tg-C in 2004 and then reaching 1,112 Tg-C in 2018 (Fig 4).

The reduction of TCS is due to the decreased extent of the natural areas (that is, forest and grassland formations, which decreased by 38% and 55%, respectively), partially offset by an increase of agricultural areas, especially rainfed agriculture (265%) and pastureland (40%) (Fig 4, Table 1). Although both AGB and BGB contribute to the reduction of total C storage, the highest C loss was from the soil reservoir; SCS_100_ accounts for about 80% of TCS and is nine times greater than AGB and seven times greater than BGB (Table 7).

## Discussion

### Soil carbon stocks

This study presents soil C stocks data to depths of 30 cm, 60 cm, and 100 cm for different LUs and LCs of Western Bahia. It is the most comprehensive collection of soil carbon stocks currently available. However, the uncertainty of our results is still high. The high level of uncertainty in our SCS estimates is related both to the dependence of SCS on biophysical factors throughout the Cerrado landscape and to the small number of subsamples taken from each site. Biophysical factors such as the degree of Cerrado preservation, amount of total accumulated precipitation, fire occurrence, and elevation are some of the factors that can affect carbon stocks in areas of a given vegetation physiognomy as a result of their varied effects on net primary production and, consequently, litter production and organic matter accumulation in soils. Although we collected soil samples from a range of LULCCs, these areas may be exposed to different environmental conditions, with consequently high variability in the results. Moreover, we have chosen areas without signs of fire or visible anthropogenic impacts, such as soil erosion or degradation, to avoid the selection of areas that have been heavily modified in recent years; this could be an additional source of variability.

Even with the high variability in our results, we demonstrate that soil C stocks are significantly affected by land use changes in two cases.

The first case is when forestlands are converted to pasturelands. For this land use transition, SCS_100_ declined by 37.3% (*p* = 0.031). Implementation of managed pastures has long been considered an activity that increases soil C stocks (for soils with > 45% clay content and for depths of 0 − 20 cm) when compared with soils of native vegetation areas due to pastures’ higher net primary production and contributions to soil structure maintenance [56–59]. However, a recent review observes that a small number of studies show relatively large soil C increases when pasture replaces native vegetation and these offset the small declines described in the majority of the studies, suggesting that soil C increases on average [60]. According to the authors, there are more studies showing that soil C declined rather than increased when pasture replaced native vegetation. Although their study reports losses of 0.13 Mg-C ha^−1^ yr^−1^ for tropical regions, no sample for the sandy Cerrado soils was used. Here, we have shown some evidence that the replacement of forests with pasture is a soil carbon source in Western Bahia.

The PAST SCS_30_ estimate (28.0 Mg-C ha^−1^ ± 11.4 Mg-C ha^−1^) is similar to estimates by [61], which found 28.87 Mg-C ha^−1^ ± 9.23 Mg-C ha^−1^ for a managed pasture in a sandy soil, which is higher than values collected at degraded pasture areas (23.87 Mg-C ha^-1^ ± 5.67 Mg-C ha^-1^). The use of soil samples collected in different types of forage grasses (such as *Brachiaria*, *Panicum*, and *Andropogon*) and the choice of sampling seasons used to estimate SCS_100_ in PAST are among the factors affecting the soil C estimates [62]. For example, during the dry season, all these forage grass systems have lower amounts of soil C (0− 20 cm) when compared with the wet season, and different grasses have different strategies for partitioning carbon content into high- and low-lability soil carbon fractions.

The low SCS values in PAST areas compared with CDO and FOR areas result from losses due to overgrazing, lack of annual maintenance fertilization (liming and application of nitrogen and phosphorus), and inadequate management (as evidenced by the presence of termite mounds and erosion processes). In sandy Cerrado soils, the acidity and low fertility quickly decrease the amount of AGB under grazing and a lack of maintenance fertilization results in low levels of soil organic carbon. In addition, unlike the rainfed croplands in Western Bahia, most of the pastureland sites we visited are not renovated every four years but are instead neglected: the extensive pastureland areas provide a large space to regrow grasses and many farmers use them until complete pasture depletion.

In the second case, SCS are affected by agricultural management when irrigated croplands replace rainfed croplands or pasturelands. The implementation of irrigation on rainfed agriculture areas is a practice that is extremely likely to increase SCS. According to [16], the irrigated croplands in sandy soils of Western Bahia can accrue 2.6% per year in soil carbon content and may in the future exceed the amount of carbon in Cerrado soils for the 0–20 cm layer. Here, our estimates show that compared with RAG, irrigated agriculture increased SCS_30_ by 28% (*p* = 0.018) and SCS_100_ by 26.5% (*p* = 0.034). The higher SCS_100_ in IRR areas is related to higher water availability and the production of two to three crops a year when compared with RAG areas, yielding higher annual net primary production. These measurements, however, may have been affected by the fact that irrigated samples were collected in the middle of the crop cycle, when crops cover the whole soil surface and reduce the soil temperature, which is unfavorable to organic matter decomposition.

Although irrigated agriculture shows potential as a carbon dioxide removal activity, apparently, SCS is high because carbon input to the soil is high, not necessarily because soil carbon is resilient. Thus long-term total carbon storage potential is unknown.

Moreover, we have evaluated the SCS data as if they were in equilibrium. In the literature, evaluation of soil carbon history indicates that no-tillage practices and well-managed pasture implementations in Cerrado soils with more than 50% clay content may increase SCS in the long-term [63–65]. For example, [66] reported increases in soil C of 34%, 47%, and 61% for the 0 − 40 cm layer when no-tillage practices were applied over two, four, and six years, respectively. For pasturelands throughout the world, it is known that improved grazing management, fertilization, irrigation, and sowing legumes and improved grass species tend to increase soil C at rates ranging from 0.1 to more than 1 Mg-C ha^−1^ yr^−1^ [60]. The first soil C accumulation rates for Western Bahia were reported by [16] who, using data from 2010–2018, found increases in soil C of 3% year^−1^ for rainfed agriculture in soils with more than 25% clay content (*p* < 0.01) and non-significant changes for soils with less than 25% clay content.

Due to the intrinsic physical and structural characteristics of sandy soils, it is important to monitor C stocks over time and to evaluate the resilience of accumulated C estimates. Crop rotation, no-tillage [6,67], reduced tillage, irrigation, increased belowground inputs [15], and forage grass type [62] are factors that affect C accumulation in the long term. However, it remains to be determined whether this behavior is also observed in sandy Cerrado soils. In Western Bahia, farmers commonly use mixed tillage in rainfed and irrigated croplands to avoid annual soil overturning, aiming to reduce C losses and increase soil C. Although many of these farmers claim that this practice contributes to increased soil C stocks over time in sandy Cerrado soils, we do not have long-term data to support this. Monitoring soil C stocks and increasing soil carbon sampling for these sandy soils are essential for understanding the soil C behavior and proposing sustainable agriculture alternatives.

Our results provide observed data that can be used to train or validate digital soil maps and expand SCS mapping to all sandy soils of the Cerrado domain. Digital soil mapping has been used to upscale the observed data from local or regional to national scales through machine learning algorithms (such as random forests [68,69], kriging-based models [70,71], and generalized linear model boosting [28]). The upscaled data are valuable for developing accurate methods to estimate soil carbon stocks and baselines in support of achieving sustainable development goals and assessing effects related to climate changes [8].

### Spatial and temporal patterns of carbon stocks for Western Bahia

Reconstructing historical carbon stocks allowed us to compute Western Bahia’s carbon balance over 28 years. We estimate that the region lost 61.9 ± 2.20 Tg-C between the periods 1990–1996 and 2011–2018, corresponding to a non-significant average change of 4.62 Mg-C ha^−1^ (*p* = 0.725) across the 131,168 km^2^ of Western Bahia.

The soils are the major contributor to total C losses (80%), losing 48.8 ± 9.81 Tg-C of total C storage between the periods 1990–1996 and 2011–2018, followed by the losses in BGB (9.37 ± 7.87 Tg-C) and AGB (3.70 ± 8.96 Tg-C).

Given the high uncertainties involved in the estimates of AGB, BGB, and SCS and the assumption of equilibrium for these carbon pools, these estimates must be interpreted as rough and should be used carefully to determine alternatives that minimize carbon losses by land use change. [65] published long-term estimates of SCS for a red-yellow latosol with clay content ranging from 50% to 70% in the Cerrado domain and demonstrated that soil C stocks in rainfed agriculture under a no-tillage system when they reached the same levels as natural Cerrado soils. Unlike [65], our data are restricted to a single annual sampling and were collected mainly from sandy soils, which by their nature are less able to protect organic matter in soil aggregates and clay minerals and therefore have a lower maximum soil carbon storage capacity compared with clayey soils.

Although we do not have data to determine how different our SCS_100_ values are from the maximum soil C storage capacity in the sandy domain of the Cerrado, we suggest that the maximum soil C storage capacity is higher than or equal to the amount of SCS_100_ in forest formations (typically *Cerradão* and gallery forests). In these areas, independent of soil textural and pedogenetic characteristics, there is high net primary production compared with production in other LUs, favoring the soil biota. Given the high water availability, the SCS_100_ in these areas and in irrigated croplands may be closer to maximum soil C storage capacity than in other areas that experience seasonal drought.

As discussed before, one of the best alternatives for reducing soil C emissions is to avoid the replacement of forest formations with pasturelands and to conserve these forests. Currently Western Bahia has 2 Mha of forestlands storing 171 Tg-C in SCS_100_ and 213 Tg-C when considering all carbon reservoirs (Table 6). In addition, maintaining natural vegetation cover in the Cerrado domain strongly contributes to the resilience of rainfed agriculture through water recycling by evapotranspiration [72] and to conservation of biodiversity.

Another option for increasing soil C stocks and reducing soil C emissions is the replacement of pasture and rainfed agriculture with irrigated agriculture. However, this solution could have severe economic, social, and environmental impacts regarding water and environmental conservation. Although on one hand the replacement of grazing lands with irrigated cropland may improve soil quality conditions, thus increasing SCS, on the other hand it raises concerns about water use. In Western Bahia, water demand for irrigation has instigated local water conflicts and prompted major concerns regarding the management and conservation of water resources [73]. Although the region is partially located on the Urucuia aquifer and is drained by three important rivers, water availability will be a limiting factor for irrigation expansion if not carefully managed. The amount of water available for consumptive use by irrigation is directly linked to the streamflow of rivers, which depends on precipitation rates, duration of the wet season, maintenance of natural vegetation cover, and recharge rates of the aquifer. Although the changes in land cover do not significantly affect soil water infiltration in Western Bahia [40], it has been demonstrated elsewhere in the Cerrado that such changes may affect the water cycle [72]. Further studies specific to this region are needed.

Moreover, the expansion of agriculture in Western Bahia is intrinsically related to landscape characteristics and availability of environmental resources. For example, in Formosa do Rio Preto (Fig 1), where the terrain is predominantly flat, there was an expansion of rainfed agriculture, including land for soybeans, cotton, and beans [74]. Meanwhile, in the Angical region (Fig 1), where natural seasonal forest predominates, pasture is the main agricultural land use, since RAG or IRR practices are infeasible due to the rugged landscape.

Evaluating the feasibility of implementing rainfed or irrigated agriculture in areas where the pasture is degraded or abandoned may be an efficient way to identify priority areas to increase SCS. If we imagine that all PAST areas (1.51 Mha) in Western Bahia are transformed into RAG and that the soils reach the average SCS levels measured in rainfed agriculture areas (57.4 Mg-C ha^−1^), then 8.62 Tg-C would be gained by soils—an average of 5.7 Mg-C ha^−1^. These values may be even higher if the farmers adopt soil conservation management practices. Crop rotation, tillage reduction, restitution of crop residues, and soil fertilization are management options that allow an increase of SCS at the local scale [50,51], while contributing to soil fertility and food security. Imagining the replacement of all PAST (1.51 Mha) and RAG areas (3.08 Mha) with IRR, the gain in SCS could be between four and seven times higher than the SCS_100_ increases achievable by replacing all PAST areas with RAG, with values reaching 39.9 Tg-C and 63.7 Tg-C, respectively.

We are still far from answering certain questions that will allow us to propose effective measures to reduce soil C losses. For example, which agricultural management practices would be best to implement to increase soil C stocks in sandy soils? And at what rate and how long would it be necessary to use them to compensate for carbon emissions due to land use change? Nevertheless, we report here a state-of-the-art study of soil C stocks in sandy soils of the Cerrado domain, where areas of rainfed agriculture and pasture seem to be acting as sources of carbon to the atmosphere. The use of irrigation is a potential option for mitigation of emissions, but the capacity is not large enough to offset emissions due to deforestation; this result affirms that avoiding deforestation is still the best option for reducing carbon emissions.

However, as previously discussed, it is necessary to improve the robustness and increase the confidence of these estimates to determine the actual impacts of land use change on carbon balance. Moreover, there is an urgent need to evaluate the lability of soil C stocks in order to determine management practices with a higher chance of effectively mitigating soil C emissions in Western Bahia. The need for long-term studies to obtain more data on transient (non-equilibrium) AGB, BGB, and SCS to contribute to the studies of carbon dynamics in sandy soil domains continues to be a challenge.

## Conclusion

This study shows that it is very likely that conversion of forestlands to pasturelands in the sandy soils of Western Bahia decreases soil carbon stocks and that the introduction of irrigation practices in rainfed croplands or pasturelands increases soil carbon stocks in the top meter of soil (*p* < 0.05). All other changes in LC and LU, such as changes from natural vegetation areas (forestlands or Cerrado formations) to rainfed agriculture and from Cerrado formations to irrigated croplands, led to non-significant changes in soil carbon stocks (*p* > 0.05). This low significance, however, may be judged in relative terms, given the high uncertainty associated with the soil carbon stocks measured. Despite the high uncertainty, this is the first estimate of the role of sandy Cerrado soils as carbon sources or sinks.

With respect to historical changes, we estimated that land use changes caused loss of 5.28% (61.9 Tg-C) of the total carbon stock in Western Bahia between the periods 1990–1996 and 2011–2018, although this result was not statistically significant. This change is strongly associated with the land use changes occurring from 1990 to 2018, including the increase of pasture areas by 40%, rainfed agriculture by 265%, and irrigated areas by 1000%. Soils were the major contributor to total carbon losses, losing 48.8 Tg-C, followed by losses from BGB (9.37 Tg-C) and AGB (3.70 Tg-C). Monitoring carbon stocks over time is important for reducing the uncertainty of carbon balance measurements and identifying appropriate management practices and time scales for changing the carbon loss trend in the sandy soil domain of the Cerrado agricultural frontier.

Given the information currently available, we conclude that the best way to avoid soil carbon losses from the sandy soils of the Cerrado is to maintain the natural forest cover and encourage farmers to apply sustainable management practices to increase and maintain the soil carbon stocks, especially in pasturelands. Although farmers have been looking for alternatives to solve the challenge of soil carbon loss in Western Bahia by using a crop rotation system, a mixed planting system, or high-technology methods to avoid soil compaction, this study demonstrates that the challenge remains.

## Supporting information

S1 Table Additional information of experiment samples. Geographic coordinates, and organic soil carbon for depths 0-5, 5-10, 10-15, 15-20, 20-40, 40-60, and 60-100 cm, for each sampling point.
(DOCX)
