# Supplementary material for: Carbon stocks and dynamics of different land uses on the Cerrado agricultural frontier
Source: PLoS One. 2020 Nov 6;15(11):e0241637. doi: 10.1371/journal.pone.0241637 (PMC7647089; doi:10.1371/journal.pone.0241637)
Supplement: S1 Table — Geographic coordinates, and organic soil carbon for depths 0-5, 5-10, 10-15, 15-20, 20-40, 40-60, and 60-100 cm, for each sampling point. (DOCX) [file pone.0241637.s001.docx]

**S1 Table. Additional information of experiment samples.** Geographic coordinates, and organic soil carbon for depths 0***–***5, 5***–***10, 10***–***15, 15***–***20, 20***–***40, 40***–***60, and 60***–***100 cm, for each sampling point.

| LULCC |  |  | Organic carbon dag kg^-1^ | | | | | | |
| --- | --- | --- | --- | --- | --- | --- | --- | --- | --- |
|  | Latitude | Longitude | 0-5 | 5-10 | 10-15 | 15-20 | 20-40 | 40-60 | 60-100 |
| SF | 12°09'S | 45°22'W | 0.06 | 0.14 | 0.06 | 0.06 | 0.06 | 0.14 | 0.14 |
| SF | 11°55'S | 45°26'W | 0.23 | 0.31 | 0.23 | 0.31 | 0.40 | 0.14 | 0.06 |
| SF | 11°51'S | 45°45'W | 0.75 | 0.93 | 0.84 | 0.84 | 0.57 | 0.23 | 0.23 |
| SF | 11°53'S | 45°50'W | 0.93 | 0.75 | 0.66 | 0.48 | 0.31 | 0.23 | 0.06 |
| SF | 10°25'S | 45°33'W | 1.51 | 1.51 | 1.51 | 1.41 | 1.22 | 0.93 | 0.66 |
| SF | 12°42'S | 44°37'W | 1.31 | 1.71 | 2.13 | 1.51 | 0.93 | 1.03 | 0.66 |
| SF | 13°03'S | 46°06'W | 0.23 | 0.31 | 0.23 | 0.14 | 0.06 | 0.14 | 0.06 |
| SF | 14°14'S | 46°03'W | 0.31 | 0.48 | 0.66 | 0.14 | 0.23 | 0.40 | 0.54 |
| SF | 14°02'S | 45°48'W | 0.66 | 0.84 | 0.48 | 0.23 | 0.23 | 0.14 | 0.06 |
| SF | 14°36'S | 45°59'W | 1.12 | 0.93 | 1.22 | 0.93 | 0.48 | 0.14 | 0.06 |
| SF | 14°23'S | 45°43'W | 0.93 | 1.22 | 1.22 | 1.12 | 0.66 | 0.23 | 0.14 |
| SF | 13°52'S | 45°20'W | 1.71 | 1.41 | 1.51 | 1.03 | 1.12 | 0.57 | 0.23 |
| SF | 13°40'S | 45°18'W | 1.03 | 0.93 | 0.93 | 0.57 | 0.66 | 0.66 | 0.57 |
| SF | 12°27'S | 44°26W | 2.35 | 2.13 | 1.81 | 1.51 | 1.03 | 0.40 | 0.75 |
| SF | 13°21'S | 45°23'W | 0.84 | 1.51 | 1.03 | 0.40 | 0.14 | 0.06 | 0.06 |
| SF | 13°46'S | 46°04'W | 0.31 | 0.14 | 0.40 | 0.40 | 0.31 | 0.48 | 0.06 |
| SF | 12°05'S | 45°02'W | 0.48 | 0.57 | 0.40 | 0.40 | 0.31 | 0.14 | 0.23 |
| SF | 11°07'S | 46°14'W | 0.75 | 0.66 | 0.93 | 1.31 | 0.93 | 0.66 | 0.57 |
| SF | 12°57'S | 45°05'W | 0.66 | 0.75 | 0.75 | 0.40 | 0.48 | 0.40 | 0.57 |
| SF | 13°02'S | 45°07'W | 0.93 | 0.66 | 0.75 | 0.66 | 0.66 | 0.31 | 0.40 |
| SF | 12°05'S | 45°00'W | 0.93 | 0.06 | 0.93 | 0.75 | 0.75 | 0.66 | 0.75 |
| FF | 11°46'S | 45°46'W | 2.46 | 1.31 | 1.71 | 1.22 | 1.12 | 0.93 | 0.75 |
| FF | 12°23'S | 44°56'W | 1.51 | 1.41 | 1.12 | 0.23 | 0.23 | 0.06 | 0.06 |
| FF | 12°25'S | 44°56'W | 1.22 | 0.93 | 0.40 | 0.23 | 0.23 | 0.14 | 0.06 |
| FF | 12°54'S | 45°29'W | 1.70 | 0.75 | 1.22 | 0.93 | 0.75 | 0.31 | 0.23 |
| FF | 12°26'S | 45°24'W | 0.75 | 0.93 | 0.57 | 0.66 | 0.66 | 0.23 | 0.14 |
| FF | 12°15'S | 45°02'W | 1.92 | 1.81 | 1.31 | 1.03 | 1.12 | 0.48 | 0.31 |
| FF | 12°39'S | 44°35'W | 1.51 | 1.22 | 1.03 | 0.93 | 0.75 | 0.40 | 0.23 |
| FF | 13°05'S | 45°59'W | 2.13 | 2.02 | 1.71 | 1.31 | 1.12 | 0.57 | 0.48 |
| FF | 13°25'S | 45°31W | 0.75 | 1.71 | 1.03 | 0.75 | 0.57 | 0.06 | 0.06 |
| FF | 12°23'S | 44°51'W | 0.69 | 0.93 | 1.12 | 0.75 | 0.48 | 0.31 | 0.48 |
| FF | 12°29'S | 44°22'W | 1.71 | 1.31 | 1.51 | 0.40 | 1.12 | 0.66 | 0.66 |
| FF | 11°54'S | 45°53'W | 2.58 | 2.69 | 1.71 | 1.22 | 1.22 | 0.84 | 0.66 |
| FF | 11°45'S | 44°10'W | 0.75 | 0.40 | 0.31 | 0.40 | 0.14 | 0.06 | 0.06 |
| FF | 11°40S | 43°59'W | 0.75 | 0.75 | 0.48 | 0.48 | 0.23 | 0.06 | 0.06 |
| FF | 11°57'S | 44°10'W | 1.31 | 1.81 | 2.02 | 1.81 | 1.41 | 0.57 | 0.23 |
| FF | 12°04'S | 44°57'W | 1.81 | 1.03 | 0.93 | 0.48 | 0.40 | 0.57 | 0.40 |
| FF | 11°59'S | 44°55'W | 0.93 | 0.48 | 0.40 | 0.31 | 0.14 | 0.23 | 0.23 |
| FF | 12°01'S | 44°57'W | 0.93 | 0.31 | 0.48 | 0.40 | 0.31 | 0.23 | 0.23 |
| FF | 11°56'S | 44°33'W | 0.93 | 0.40 | 0.31 | 0.14 | 0.23 | 0.40 | 0.40 |
| FF | 12°06'S | 44°56'W | 1.41 | 1.22 | 1.22 | 0.75 | 0.75 | 0.66 | 0.31 |
| IRR | 12°26'S | 45°17'W | 1.28 | 0.71 | 0.64 | 0.42 | 0.42 | 0.35 | 0.21 |
| IRR | 12°25'S | 45°17'W | 0.92 | 0.85 | 0.49 | 0.71 | 0.64 | 0.35 | 0.42 |
| IRR | 12°25'S | 45°16'W | 0.92 | 0.78 | 0.71 | 0.71 | 0.57 | 0.71 | 0.42 |
| IRR | 12°53'S | 45°30'W | 1.13 | 0.99 | 1.06 | 1.06 | 0.71 | 0.42 | 0.42 |
| IRR | 12°45'S | 45°15'W | 0.78 | 0.78 | 0.71 | 0.78 | 0.85 | 0.78 | 0.45 |
| IRR | 12°45'S | 45°16'W | 0.92 | 0.78 | 0.71 | 0.49 | 0.57 | 0.49 | 0.45 |
| IRR | 12°46'S | 45°18'W | 0.85 | 0.85 | 0.71 | 0.64 | 0.64 | 0.49 | 0.45 |
| IRR | 12°47'S | 45°17'W | 1.21 | 1.13 | 1.28 | 1.13 | 0.71 | 0.57 | 0.52 |
| IRR | 11°46'S | 45°37'W | 0.99 | 0.99 | 0.92 | 0.64 | 0.49 | 0.49 | 0.57 |
| IRR | 11°46'S | 45°37'W | 1.42 | 1.21 | 1.06 | 0.85 | 0.49 | 0.49 | 0.49 |
| IRR | 11°40'S | 45°45'W | 1.91 | 1.21 | 1.22 | 0.65 | 1.22 | 0.58 | 0.21 |
| IRR | 11°41'S | 45°39'W | 2.24 | 0.72 | 0.58 | 0.44 | 0.44 | 0.44 | 0.44 |
| IRR | 12°09'S | 45°51'W | 0.58 | 0.50 | 0.44 | 0.29 | 0.36 | 0.21 | 0.21 |
| IRR | 12°09'S | 45°54'W | 1.30 | 0.94 | 0.79 | 0.72 | 0.36 | 0.29 | 0.29 |
| IRR | 12°13'S | 45°55'W | 1.22 | 1.15 | 1.15 | 0.79 | 0.65 | 0.65 | 0.44 |
| IRR | 12°14'S | 45°55'W | 0.86 | 0.58 | 0.86 | 0.50 | 0.36 | 0.36 | 0.36 |
| IRR | 12°14'S | 45°57'W | 1.08 | 0.58 | 0.50 | 0.36 | 0.36 | 0.21 | 0.29 |
| IRR | 11°47'S | 45°43'W | 1.44 | 1.15 | 1.15 | 0.94 | 0.65 | 0.65 | 0.44 |
| IRR | 11°45'S | 45°43'W | 1.30 | 1.30 | 1.15 | 0.86 | 0.58 | 0.50 | 0.36 |
| IRR | 11°46'S | 45°43'W | 1.08 | 1.08 | 0.72 | 0.79 | 0.58 | 0.58 | 0.50 |
| PAST | 12°09'S | 45°24'W | 0.06 | 0.14 | 0.06 | 0.06 | 0.06 | 0.06 | 0.23 |
| PAST | 12°07'S | 45°22'W | 0.23 | 0.31 | 0.23 | 0.40 | 0.14 | 0.06 | 0.06 |
| PAST | 11°55'S | 45°25'W | 0.23 | 0.31 | 0.06 | 0.06 | 0.14 | 0.06 | 0.06 |
| PAST | 11°55'S | 45°25'W | 0.23 | 0.14 | 0.14 | 0.06 | 0.31 | 0.06 | 0.06 |
| PAST | 12°15'S | 45°02'W | 1.71 | 1.22 | 1.12 | 1.03 | 0.75 | 0.14 | 0.31 |
| PAST | 12°15'S | 45°02'W | 0.31 | 0.23 | 0.40 | 0.23 | 0.23 | 0.31 | 0.40 |
| PAST | 13°26'S | 45°33'W | 0.31 | 0.73 | 0.31 | 0.57 | 0.06 | 0.06 | 0.06 |
| PAST | 14°14'S | 46°01'W | 1.22 | 0.14 | 0.23 | 0.23 | 0.06 | 0.06 | 0.14 |
| PAST | 12°02'S | 45°48'W | 0.23 | 0.31 | 0.06 | 0.06 | 0.23 | 0.14 | 0.06 |
| PAST | 14°29'S | 45°48'W | 0.57 | 0.48 | 0.31 | 0.40 | 0.31 | 0.14 | 0.06 |
| PAST | 14°24'S | 45°43'W | 1.03 | 0.75 | 0.84 | 0.48 | 0.57 | 0.48 | 0.49 |
| PAST | 13°52'S | 45°20'W | 1.22 | 1.31 | 1.22 | 1.03 | 0.75 | 0.57 | 0.48 |
| PAST | 12°31S | 44°25'W | 2.58 | 2.13 | 2.24 | 2.13 | 1.51 | 1.41 | 0.75 |
| PAST | 13°28'S | 45°26'W | 0.48 | 0.31 | 0.14 | 0.23 | 0.14 | 0.23 | 0.14 |
| PAST | 11°53'S | 44°07'W | 0.66 | 0.48 | 0.14 | 0.31 | 0.06 | 0.06 | 0.57 |
| PAST | 11°49'S | 44°08'W | 1.03 | 0.93 | 0.31 | 0.40 | 0.14 | 0.06 | 0.06 |
| PAST | 11°27'S | 44°53'W | 1.03 | 0.93 | 0.75 | 0.57 | 0.66 | 0.48 | 0.23 |
| PAST | 11°28'S | 44°54'W | 0.75 | 0.40 | 0.31 | 0.14 | 0.06 | 0.14 | 0.48 |
| PAST | 12°09'S | 44°37'W | 0.75 | 0.40 | 0.31 | 0.40 | 0.14 | 0.23 | 0.14 |
| PAST | 12°09'S | 44°37'W | 0.23 | 0.66 | 0.48 | 0.31 | 0.14 | 0.23 | 0.14 |
| PAST | 12°57'S | 45°05'W | 0.75 | 0.40 | 0.57 | 0.48 | 0.75 | 0.48 | 0.40 |
| RAG | 11°49'S | 45°44'W | 0.48 | 0.57 | 0.23 | 0.23 | 0.14 | 0.06 | 0.06 |
| RAG | 11°58'S | 45°56'W | 0.66 | 0.66 | 0.66 | 0.57 | 0.75 | 0.57 | 0.48 |
| RAG | 12°13'S | 45°56'W | 0.66 | 0.23 | 0.06 | 0.06 | 0.06 | 0.06 | 0.06 |
| RAG | 12°12'S | 45°57'W | 0.14 | 0.31 | 0.06 | 0.06 | 0.14 | 0.06 | 0.14 |
| RAG | 10°20'S | 45°30'W | 1.22 | 0.93 | 0.93 | 1.03 | 1.12 | 0.14 | 0.23 |
| RAG | 10°25'S | 45°33'W | 1.22 | 0.93 | 0.93 | 0.75 | 0.75 | 0.57 | 0.31 |
| RAG | 12°54'S | 45°30'W | 0.31 | 0.57 | 0.23 | 0.06 | 0.06 | 0.06 | 1.12 |
| RAG | 12°26'S | 45°27'W | 0.14 | 0.66 | 0.40 | 0.48 | 0.14 | 0.23 | 0.06 |
| RAG | 12°40'S | 44°34'W | 0.75 | 0.84 | 0.93 | 1.22 | 0.93 | 0.48 | 0.23 |
| RAG | 13°06'S | 45°58'W | 0.75 | 1.22 | 0.75 | 0.48 | 0.40 | 0.48 | 0.31 |
| RAG | 13°02'S | 46°06'W | 0.93 | 0.93 | 1.03 | 0.23 | 0.48 | 0.31 | 0.06 |
| RAG | 13°03'S | 46°06'W | 0.14 | 0.14 | 0.06 | 0.06 | 0.14 | 0.14 | 0.14 |
| RAG | 13°26'S | 45°31'W | 1.22 | 1.12 | 0.75 | 0.93 | 1.22 | 0.31 | 0.31 |
| RAG | 14°14'S | 46°01'W | 0.31 | 0.06 | 0.66 | 0.31 | 0.06 | 0.06 | 0.06 |
| RAG | 14°04'S | 45°49'W | 0.14 | 0.14 | 0.06 | 0.31 | 0.06 | 0.14 | 0.06 |
| RAG | 14°36'S | 45°58'W | 1.03 | 0.93 | 0.93 | 0.40 | 0.31 | 0.66 | 0.06 |
| RAG | 14°23'S | 45°42'W | 0.75 | 0.40 | 0.48 | 0.14 | 0.23 | 0.14 | 0.06 |
| RAG | 12°27'S | 44°27'W | 1.63 | 1.61 | 1.51 | 0.93 | 0.66 | 0.57 | 0.57 |
| RAG | 13°47'S | 46°06'W | 0.66 | 0.31 | 0.23 | 0.14 | 1.03 | 0.14 | 0.14 |
| RAG | 11°06'S | 46°15'W | 1.12 | 0.93 | 0.75 | 0.23 | 0.66 | 0.48 | 0.31 |
